# Preparation of microspheres with sustained ketoprofen release by electrospray for the treatment of aseptic inflammation

**DOI:** 10.3389/fbioe.2024.1416659

**Published:** 2024-07-19

**Authors:** Xinyi Dai, Wei Nie, Chuanyou Duan, Yi Shen

**Affiliations:** ^1^ Department of Plastic and Reconstructive Surgery, Shanghai Ninth People’s Hospital, School of Medicine, Shanghai Jiao Tong University, Shanghai, China; ^2^ Wake Forest Institute for Regenerative Medicine, Wake Forest University School of Medicine, Winston-Salem, NC, United States; ^3^ Luhe People’s Hospital, Nanjing, Luhe, China; ^4^ Bio-ID Center, School of Biomedical Engineering, Shanghai Jiao Tong University, Shanghai, China

**Keywords:** electrospray, ketoprofen, shellac microsphere, aseptic inflammation, drug release

## Abstract

The treatment of aseptic inflammation has always been a clinical challenge. At present, non-steroidal drug-loaded microspheres have been widely used in the treatment of aseptic inflammation due to their excellent injectable and sustained release capabilities. In this study, ketoprofen-loaded shellac microspheres (Keto-SLAC) were prepared by electrospray. Alterations of Keto-SLAC morphology was observed in response to changed shellac concentration in ethanol solution through electrospray. Further examination revealed that ketoprofen presented as amorphous solid dispersion in the shellac microspheres. Most importantly, it was also shown that ketoprofen can be slowly released from the shellac matrix for up to 3 weeks. *In vitro* cell experiments verified that the microspheres had favorable cell compatibility. We therefore proposed that the prepared microspheres, being readily available in use in a variety of clinical settings through topical application, have promising therapeutic potential for the treatment of aseptic inflammation.

## 1 Introduction

The treatment of aseptic inflammation is a thorny issue clinically (Tanaka and Kishimoto, 2012; Tarantino et al., 2016). Nonsteroidal Anti-inflammatory Drugs (NSAIDs) are considered to be an ideal drug for the treatment of aseptic inflammation (Zhou et al., 2014). By inhibiting the production of prostaglandin and the activity of cyclooxygenase, it can effectively reduce the symptoms like fever, swelling and pain caused by aseptic inflammation (Shamsudin et al., 2018). Due to its low toxicity and high efficiency, ketoprofen has become a commonly used non-steroidal drug in the treatment of aseptic inflammation caused by arthritis and gout (Yu et al., 2018; Shohin et al., 2012). However, the systemic administration of ketoprofen is associated with some unpredictable toxicity to the liver and heart (Carbone et al., 2013). *In situ* drug delivery has therefore become a preferred route for ketoprofen administration (Stigliani et al., 2013; Basar et al., 2017).

The development of effective methods in materials science for preparing drug-loaded microspheres helps to address these shortcomings. Drug-loaded microspheres are micron or nano-sized spherical particles with drug dispersing in it. Due to the large specific surface area and high drug loading capacity of microsphere, it has been widely used for drug delivery (Andhariya and Burgess, 2016). As a matter of fact, the injectability of microspheres also allows them to be conveniently delivered to the target site (Targeted drug delivery), which only increases the concentration of the drug in the injection area but avoids unnecessary drug accumulation in other organ, thereby prevents potentially toxic effects of the drug. The common methods for preparing drug-loaded microspheres include emulsification crosslink, emulsification-heating and curing, emulsification-solvent evaporation, spray drying (Mo et al., 2016), emulsification-solvent evaporation method (Baena-Aristizábal et al., 2016) and spray drying method (Fernández-Paz et al., 2020). Although the first two methods may prepare microspheres with relatively ideal particle size distribution, the preparation flow turn out to be more complicated, in which a precise control of parameters (such as temperature, stirring speed, pH value, etc.) is required. Moreover, the use of crosslinking agents with cytotoxicity also reduces the biosafety of microspheres (Cheng et al., 2013). On the contrary, Spray-drying method is commonly adopted to prepare drug-loaded sustained-release microspheres in manufacturing industry. However, the traditional spray drying method often requires very complicated equipment, meanwhile, it also contains high temperature processing step, which will easily destroy chemical structure of bioactive molecules and polymers as drugs (Wei et al., 2020). Therefore, it is necessary to develop a simpler and safer method for the preparation of drug-loaded microspheres.

As a new method of preparing microspheres, electrospray has attracted widespread attention in recent years, as microspheres prepared by this method has wide applications such as drug delivery (Tanhaei et al., 2021), tissue engineering (Xue et al., 2020), composite materials (Xi et al., 2019) chemical sensing (Cho et al., 2020). As a relatively simple method, the main equipment needed for electrostatic spraying are injection pump, high voltage DC power supply and collector. Briefly, The polymer solution is expelled from the pump, and under a high-voltage electric field, it splits into many small droplets. The solvent in the droplet is evaporated and dried at room temperature and at last, microspheres was fall on the receiver. Compared with traditional spray drying technique, electrospray is cost-effective and suitable for most polymers (Zhang et al., 2021). Currently, a variety of polymers have been used for the preparation of electrosprayed microspheres. For example, Richard et al. combined electrostatic spray and thermally induced phase separation technique to prepare porous poly (lactic-co-glycolic) acid (PLGA) (Malik et al., 2016). Also, Tatsuo et al. reported their approach to prepare chitosan/calcium phosphate composite microspheres by electrostatic spray (Yunoki et al., 2014). However, some shortcomings of fabricated microspheres using some of the polymers are obvious: polyester microspheres are prone to produce acidic degradation products that induce inflammatory response of the host (Zhu et al., 2015). The molecular interaction of chitosan is relatively large, and toxic solvents are needed in the electrostatic spraying process (Bilican et al., 2020). Shellac, as a natural polyester with a small molecular weight, has been widely used as matrix material for drug delivery. Particularly, as it is easily soluble in ethanol, shellac turns out to be an ideal material for the preparation of microspheres by mean of electrostatic spray (Wang et al., 2018).

In view of the above, ketoprofen-loaded shellac microspheres (Keto-SLAC) was prepared by electrospray for the treatment of aseptic inflammation. The morphology as well as the physical and chemical properties of the microspheres under different spray parameters was investigated. In addition, the drug release profile and *in vitro* cell compatibility of ketoprofen-loaded microspheres have also been studied to assess its possible application against aseptic inflammation.

## 2 Materials and methods

### 2.1 Materials

All chemicals used in the experiment were purchased from Sigma-Aldrich unless stated otherwise. The experimental water was ultrapure water filtered by Mill-pore (the resistivity was 18 MΩ/cm).

### 2.2 Characterization

The surface morphology of the microspheres was observed by scanning electron microscope (SEM) (JSM 5800, Jeol Ltd., Tokyo, Japan). The thermal analysis of the microspheres is performed by DSC scanning (TA MDSC 2910, TA Instruments 10, United States °C/min, temperature range 20°C–200°C). The structure of the microspheres was further studied by XRD (Riga D/Max-BR, Tokyo, Japan, diffraction angle 4°–50°, 40 mV, and 300 mA).

### 2.3 Preparation of Keto-SLAC

In order to study the influence of different polymer concentrations on the morphology of microspheres, shellac/ketoprofen solutions of different concentrations were prepared. The ratio of ketoprofen to shellac is maintained at 1:10. Taking 2.5% shellac/ketoprofen as an example, 2.5 g of shellac was dissolved in 10 mL of ethanol. Then 0.25 g of ketoprofen was added. A transparent solution was obtained after 1 h stir (300 rpm) in a water bath at 45°C. The prepared solution was poured into a 5 mL syringe. The 18G flat needle is used as a jet capillary to connect to the positive terminal of the power supply. The aluminum foil connected to the negative electrode used as a receiving plate. The process parameters of electrospray are: the flow rate is 3 mL/h. The distance between the receiving plate and the spinneret is 15 cm and 30 cm respectively. The voltage is 10 kV, the ambient temperature is 25°C, and the ambient humidity is 40% ± 5%. The composition of different concentrations of spray liquid is listed in Table 1.

**TABLE 1:** Composition of spinning solution.

Shellac/ketoprofen solution	The mass ratio of ketoprofen to shellac (wt/wt)	Shellac concentration in ethanol solution (%)
G1	1:10	25
G2	1:10	35
G3	1:10	45
G4	1:10	55
G6	1:10	65

### 2.4 *In Vitro* drug release studies

Ketoprofen release test refers to the method published previously (Park et al., 2012). Briefly, 500 mg of Keto-SLAC microspheres were added to the dialysis bag (cut-off molecular weight is 1,000 Mw). The dialysis bag was then immersed in 100 mL of PBS solution and placed on a shaker at 37°C with speed at 300 rpm. 1 mL of sustained-release solution was drawn at different time points, and the concentration of ketoprofen was quantified by liquid chromatography (Park et al., 2012). 100 μL of fresh PBS were added to the sustained-release system after each extraction in order to balance the volume of the release solution system.

### 2.5 Cell culture

NIH 3T3 cells were purchased from ATCC (CRL1658). According to the supplier’s instructions, DMEM high glucose medium containing 10% fetal bovine serum (FBS) was used to culture 3T3 cells. Incubation was conducted at 37°C in 5% carbon dioxide. The cells are changed every 2 days during the culture. When 80% confluence is reached, the cells are passaged.

### 2.6 *In vitro* biocompatibility

The toxicity of microspheres on fibroblasts and cell viability were determined by CCK-8 and LDH tests. In short, NIH 3T3 was seeded in 96-well plates at a density of 4,000 cells/well. After incubating for 12 h, the medium was aspirated and replaced it with fresh medium with various concentrations of Keto-SLAC. The Keto-SLAC solution concentrations at 10, 20, 40, 60, 80, 160, and 320 μg/mL were co-cultured with the cells for 24 and 48 h, respectively. Then CCK-8 and LDH kits were used to detect the cell viability. All tests were performed according to the supplier’s instructions, and the cells in the untreated group were expressed as a normalized percentage.

### 2.7 *In vivo* experiment

All animal experiments were performed according to the protocols approved by the Institutional Animal Care and Use Committee at Shanghai Jiao Tong University (202401131). Wistar rats (male, 125∼150 g, 3 weeks old, n = 18) were purchased from Sino-British Sippr/BK Laboratory. Shanghai Animal Co., Ltd., China. All rats were housed under standard laboratory conditions at 25°C. During the experiment, distilled water and commercially available food (Sino-British Sippr/BK Experimental Animal Co., Ltd.) were given freely, and the animals were housed in groups in plastic bottom cages covered with sawdust. The lighting time of the breeding room is 12 h (7:00 a.m. to 7:00 p.m.). All experiments were approved by the Animal Experimentation Ethical Review Committee of Shanghai Jiao Tong University. Experimental arthritis was induced in rats as follows: each rat was injected into the joint with 0.1 mL of CFA containing a mineral oil suspension of *Mycobacterium* butyricum (Sigma Chemical Co.). After 2 weeks of CFA treatment, each rat in the drug test group (6 rats in each group) was given a single dose of prepared ketoprofen suspension,lac-ketoprofen microspheres, and naked lac at the joints according to body weight. Microspheres, dose 20 mg/kg. The control group (n = 6) was injected with the same volume of PBS. After the experiment, the rats were sacrificed, and the joints of the fore and hind limbs were removed for pathological section analysis.

### 2.8 Statistical analysis

Origin 9.0 (OriginLab, United States) was used for statistical analysis. Statistical significance was assessed by using one-way or two-way analysis of variance (ANOVA) and Tukey’s multiple comparison test. When *p* < 0.05, the data is considered significant. It is represented by *. When *p* < 0.01, it is represented by **. All data are shown as mean ± standard deviation.

## 3 Results and discussion

### 3.1 Preparation of Keto-SLAC by electrospray

**FIGURE 1:**
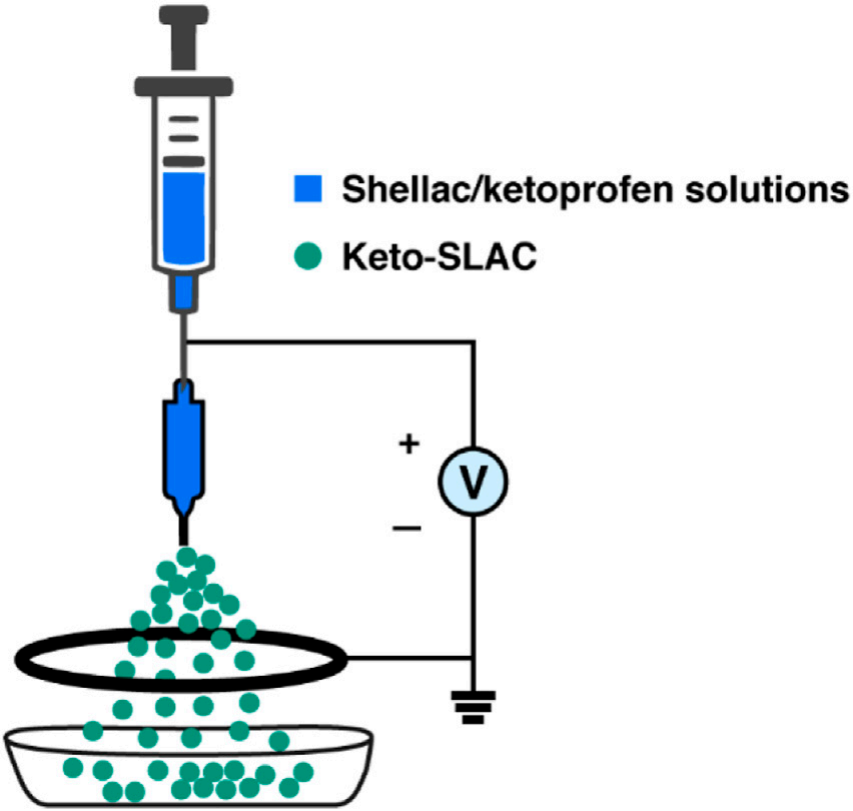
Schematic illustration of the electrospray process for Keto-SLAC fabrication.

**FIGURE 2:**
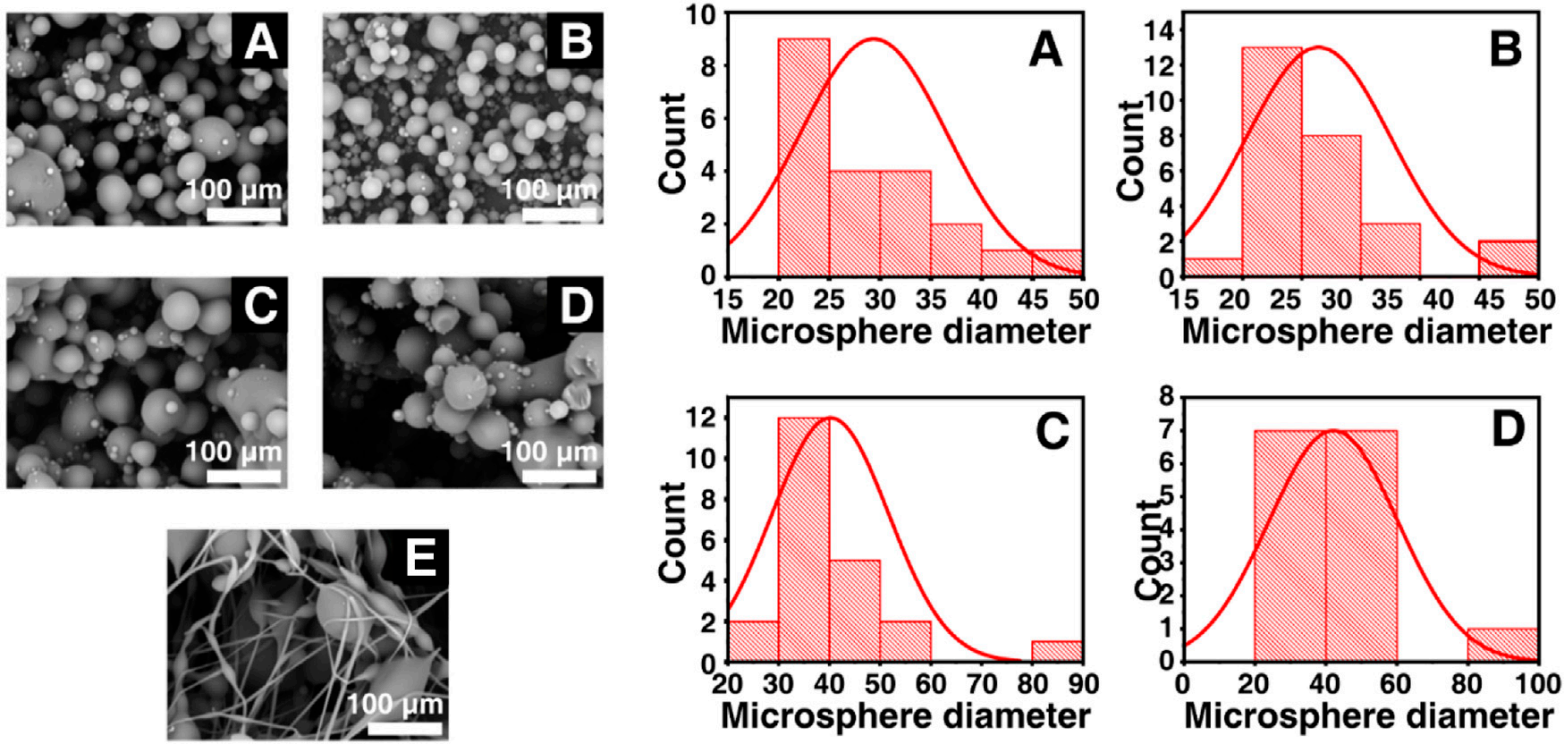
Statistical particle sizes distributions (right) of different shellac concentration derived Keto-SLAC based on SEM images (left) with the spray distance in 15 cm. **(A)** 25%, **(B)** 35%, **(C)** 45%, **(D)** 55%, **(E)** 65%.

As was shown in Figure 1, Keto-SLAC microspheres were prepared by electrospray of shellac/ketoprofen solutions of different concentrations with a spray distance of 15 cm. When the concentration of shellac is 25%–55%, the prepared microspheres have a smooth surface without agglomeration of drug particles and polymers. At the highest shellac concentration (65%), a spindle-like fiber was observed. A zoom-in picture shows the particle size distribution of the microspheres more clearly. The microspheres of all groups displayed a bimodal distribution. Small microspheres are often gathered on or around the surface of larger microspheres (Figure 2).

**FIGURE 3:**
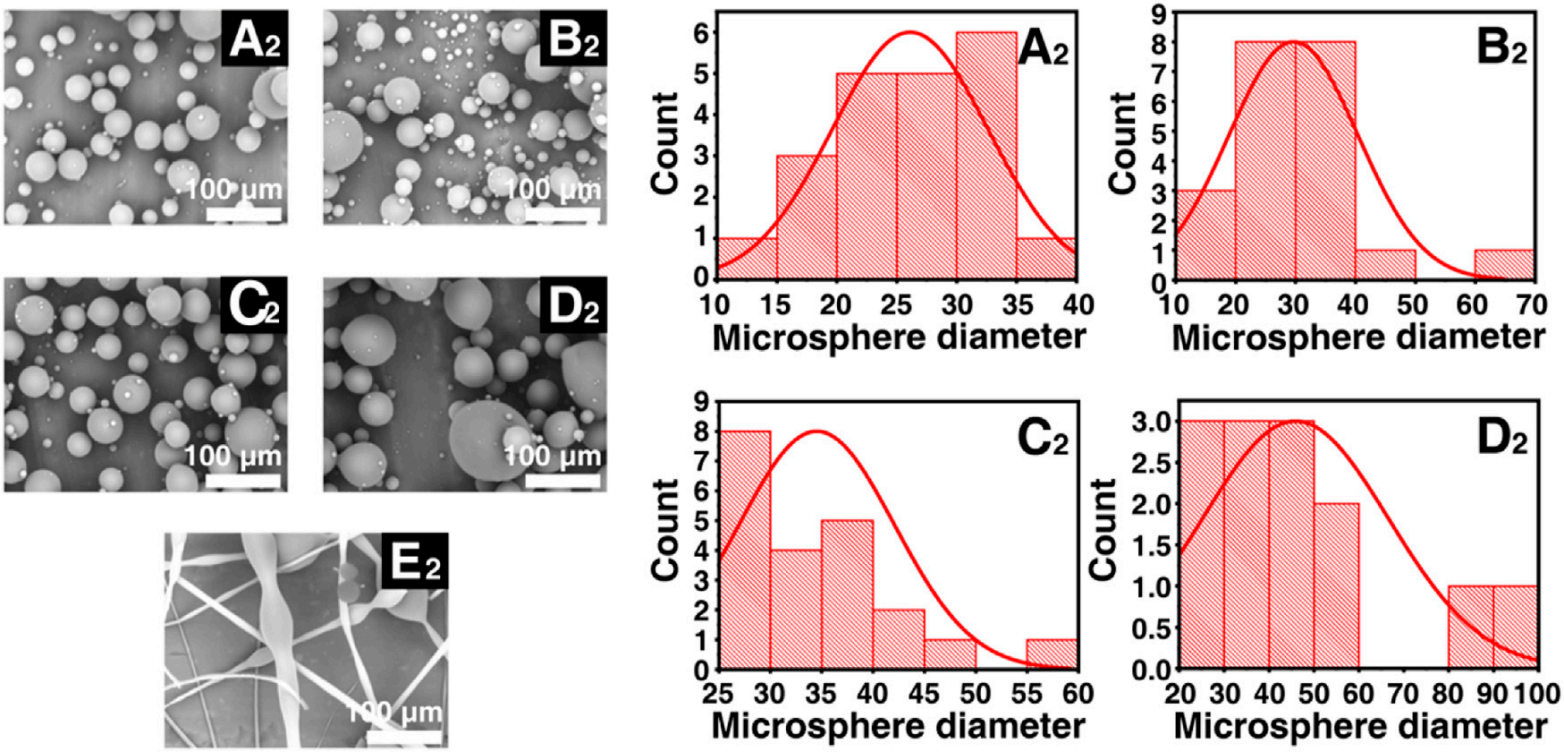
Statistical particle sizes distributions (right) of different shellac concentration derived Keto-SLAC based on SEM images (left) with the spray distance in 30 cm. **(A**_**2**_ **)** 25%, **(B**_**2**_ **)** 35%, **(C**_**2**_ **)** 45%, **(D**_**2**_ **)** 55%, **(E**_**2**_ **)** 65%.

On the other hand, particle sizes distributions of Keto-SLAC microspheres prepared under the spray distance of 30 cm was also investigated. As was shown in Figure 3, higher concentration of SLAC (65%) also results in spindle-like fiber structure. This suggested that the concentration of shellac is of critical importance for the formation of microspheres. Meanwhile, as the spraying distance increases, the particle size distribution of the microspheres becomes more uniform. It is worth noting that in the 25% drug-loaded shellac microsphere group, the particle size of the microspheres is the most uniform. These results indicated that the optimal parameter to obtain microspheres with uniform size distribution is a receiving distance at 30 cm and a shellac concentration of 25%, which brings about a resultant particle size of 27 μm in average. Therefore, Keto-SLAC microspheres prepared under such parameter are used in the follow experiments. The electrospray technique used in this study can be adapted for other drugs by adjusting parameters such as solvent type, polymer concentration, and flow rate to optimize drug encapsulation and release profiles. This method’s versatility also allows for the encapsulation of multiple drugs, making it suitable for combination therapies targeting various diseases.

### 3.2 Characterization of Keto-SLAC

**FIGURE 4:**
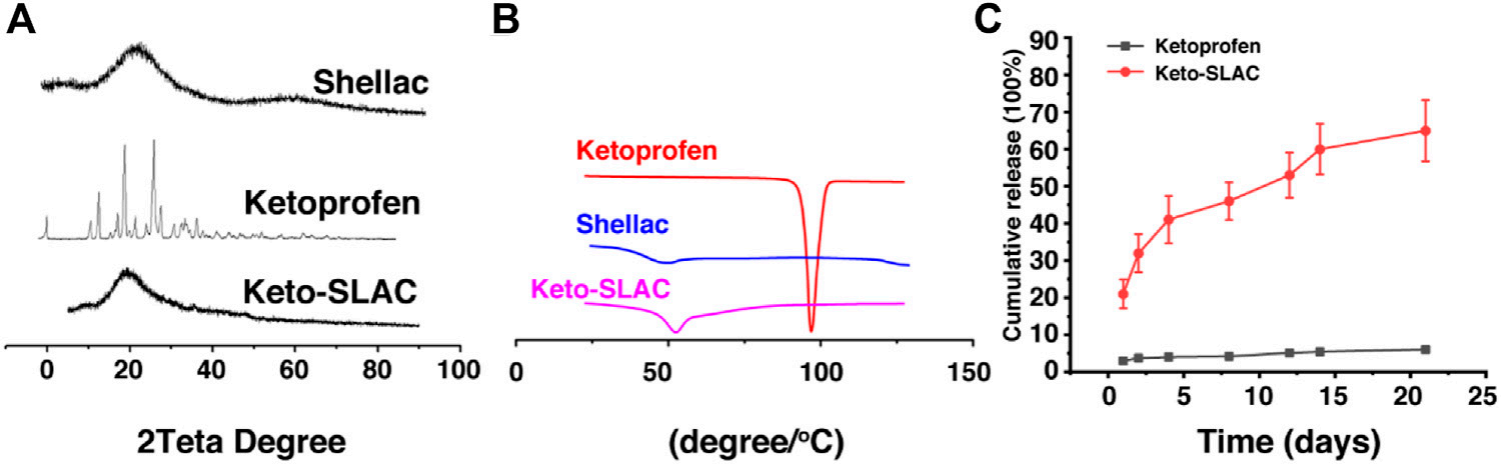
Characterization of Keto-SLAC. **(A)** XRD pattern, **(B)** Differential scanning calorimeter, **(C)** Drug release profiles.

The XRD analysis, shown in Figure 4A, indicated that there was no sharp peak in shellac spectrum, which provided evidence that there was disordered molecule arrangement with in shellac. However, several sharp diffraction peaks was seen in XRD pattern of ketoprofen, which was in consistent with the result from DSC analysis for ketoprofen, indicating an ordered crystal arrangement of ketoprofen molecule. However, crystal peak of ketoprofen disappeared in the diffraction pattern of Keto-SLAC, verifying that ketoprofen had lost its crystal structure and existed in an amorphous state in shellac microspheres. Results from both XRD and DSC provided evidence that ketoprofen had changed its physical state from crystal to amorphous solid dispersion in shellac.

Differential scanning calorimetry (DSC) analysis of the microspheres was shown in Figure 4B. It can be seen that ketoprofen has a very sharp absorption peak at 98.2°C, indicating that ketoprofen exists as an ordered crystal. There is a gentle endothermic peak around 48.3°C of shellac sample, which is the glass transition temperature of shellac. Keto-SLAC has a gentle endothermic peak at 52.6°C, which is slightly deviated from the peak of shellac. This may possibly due to the interaction of shellac and ketoprofen in the composite material. Most importantly, the endothermic peak of ketoprofen at 98.2°C disappeared in the Keto-SLAC sample, indicating that ketoprofen has lost its original crystal form and exists in the microspheres in an amorphous state.

We further examined the release profile of Keto-SLAC *in vitro*, shown in Figure 4C, in which Keto-SLAC exhibited an obvious sustained-release, which is consistent with previous reports on microspheres (DeJulius et al., 2021). From the release curve, the entire drug release lasted about:20 days, in which a relatively fast release was seen in the first 2 days, and then gradually slow down. Probably, the initial rapid release occurred as a result of ketoprofen on the surface of the microspheres. When ketoprofen on the surface of the microspheres has completely unleashed, those in the microsphere is responsible for the slow-down of drug release that gradually reached equilibrium.

### 3.3 *In vitro* cytocompatibility assay

**FIGURE 5:**
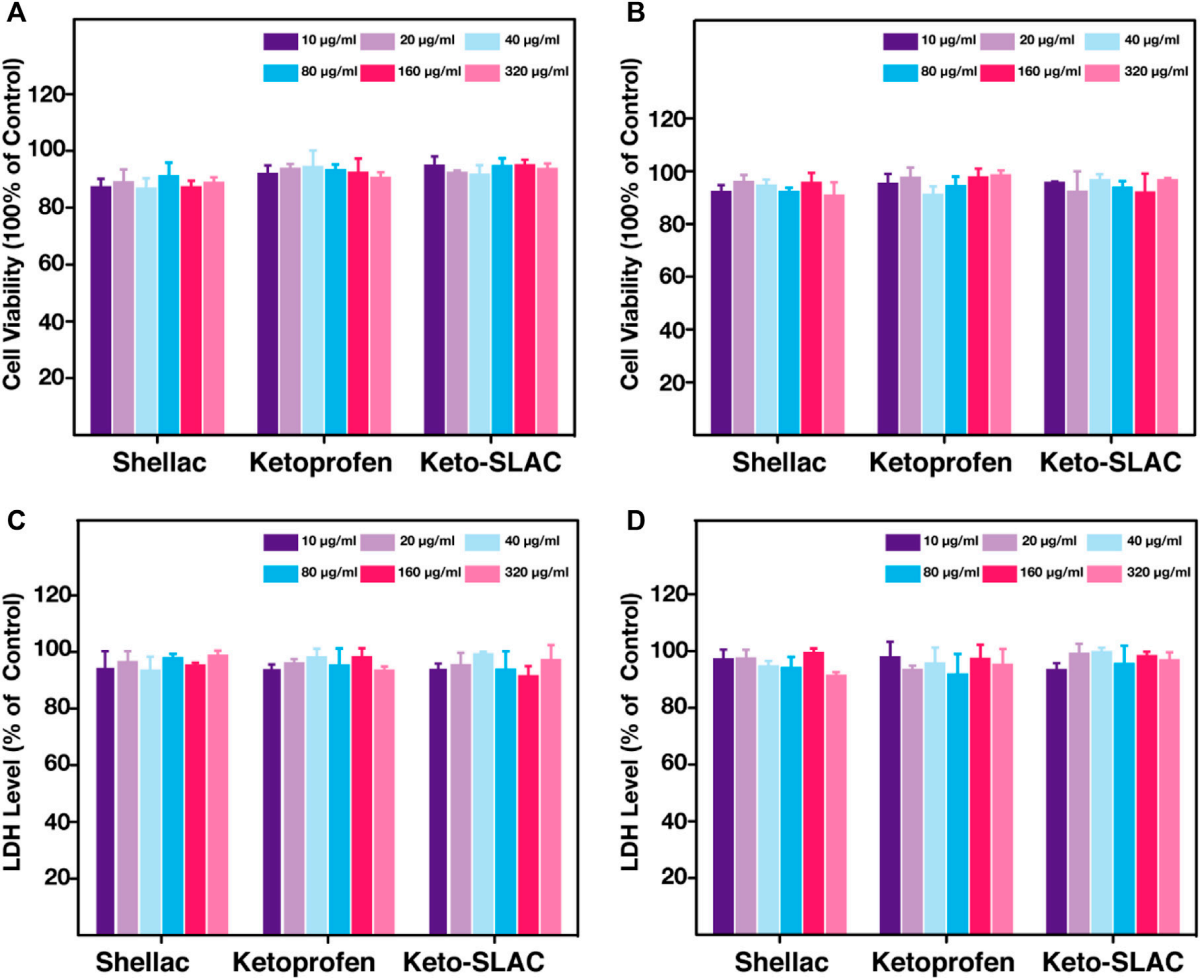
CCK-8 analysis of 3T3 cell after treatment of increasing concentration of shellac, ketoprofen and keto-SLAC for 24 h **(A)** and 48 h **(B)**. LDH test of the 3T3 cell with the same treatment as CCK-8 for 24 h **(C)** and 48 h **(D)**. No significant LDH release was found in each group.

Cell compatibility of drug-loaded microspheres was examined and shown in Figure 5, where the metabolic activity of cells subjected to Keto-SLAC of different concentrations at different time point (24 and 48 h) was measured. As compared to the untreated cells, no significant difference in cell viability was seen in cells treated with different concentrations of Keto-SLAC (Figures 5A, B). Similarly, after being treated with different concentrations of Keto-SLAC, no significant increase of LDH release was found as compared to those untreated cells, suggesting that Keto-SLAC has favorable cell compatibility and low cytotoxicity (Figures 5C, D). Previous studies have demonstrated the low cytotoxicity and favorable biocompatibility of shellac when used as a matrix material for drug delivery systems. In our study, the cell viability assay (CCK-8) and LDH release assay showed that the Keto-SLAC microspheres had no significant cytotoxic effects on NIH 3T3 cells, even at high concentrations, corroborating these previous findings. This suggests that shellac, as a natural and biodegradable polymer, is a biocompatible and safe material for drug delivery applications.

### 3.4 Treatment effect of Keto-SLAC *in vivo*

**FIGURE 6:**
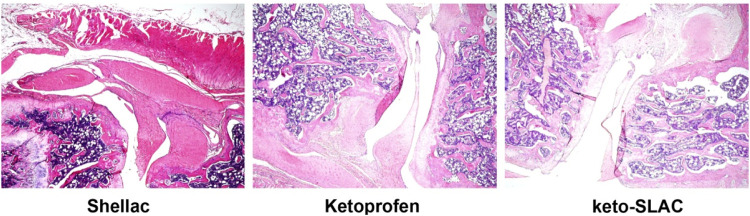
Treatment effect of Keto-SLAC on aseptic inflammation induced in a rat model.

In order to evaluate the pathological changes of the joint, H&E staining was performed after the treatment. Histological results showed that the single-injection ketoprofen treatment group still had obvious inflammatory cell infiltration, pannus formation, cartilage destruction, and synovial hyperplasia (Figure 6). In the ketoprofen microsphere treatment group, intra-articular pannus formation and inflammatory cell infiltration were significantly reduced, but visible cartilage destruction and synovial hyperplasia still existed. AA rats treated with ketoprofen microspheres had significant beneficial effects in inhibiting inflammatory cell infiltration, synovial hyperplasia, cartilage destruction, and pannus formation compared with the ketoprofen group alone. Our results indicate that ketoprofen sustained release from shellac microspheres can reduce pannus formation and significant inflammatory cell infiltration in joints, although it does not prevent cartilage destruction.

## 4 Conclusion

Ketoprofen-loaded shellac microspheres were successfully prepared by electrospray. Keto-SLAC with more uniform size is obtained by spraying 45% shellac solution at a distance of 30 cm. The DSC analysis revealed that the sharp absorption peak of ketoprofen disappeared in the microspheres. Results from XRD spectroscopy also indicated that the crystal structure of ketoprofen was disrupted as the microspheres formed. The consistent results from DSC and XRD justified the existence of solid dispersion of ketoprofen in shellac. Moreover, *in vitro* release experiments demonstrated that Keto-SLAC can be persistently released for around 20 days. Cell experiments provided evidence that the prepared microspheres had favorable compatibility *in vitro*. In addition to its favorable biological properties, shellac is a natural and biodegradable polymer, making it an environmentally sustainable choice for pharmaceutical applications. Its use in drug delivery systems aligns with the growing emphasis on reducing environmental impact and promoting sustainability in the pharmaceutical industry. In short, the application of Keto-SLAC microspheres prepared by electrospray may be a promising approach for the treatment of aseptic inflammation. Compared to current commercial drug delivery systems, the electrospray-prepared Keto-SLAC microspheres offer improved control over particle size and drug release profiles. This method also avoids high temperatures and toxic solvents, enhancing drug stability and biocompatibility.

## Data Availability

The original contributions presented in the study are included in the article/Supplementary Material, further inquiries can be directed to the corresponding authors.
